# Gripped by Destructive Forces and Threatened with Disruption of Their Organisations

**Published:** 1918-07-20

**Authors:** 


### THE CIVIL HOSPITALS OF AMERICA.

### "Gripped by Destructive Forces and Threatened with Disruption of their Organisations."

On June 3, 1918, a conference on hospital problems arising out of the war was held in New York. We hope to publish shortly a report of this conference. Meanwhile, it cannot fail to be of interest to our readers, and the hospital world generally, to study a letter addressed to the President of the United States on June 5 by the War Service Committee of the American Hospital Association. We have further received a resolution adopted by the Hospital Conference of the City of New York on June 17, 1918. These documents indicate with reasonable fulness the ground on which American hospital authorities declare that the civil hospitals of the United States are being gripped by destructive forces with a tendency to hospital disintegration, which is gaining momentum, and calls for a prompt remedy.

America's increasing participation in the world war has necessitated, in ever-growing measure, the use of physicians and nurses by the Navy and Army, the enrolment of young men as combatants, and the employment of men and women of all ages in war industries. It is declared that the hospitals of New York City are experiencing grave and constantly growing difficulty in maintaining working forces adequate to their needs. Unless remedial measures are instituted promptly it is urged that the enforced closing of single departments may prove a prelude to the discontinuance of the work of entire institutions. It is maintained, therefore, that the only hope of practical and adequate relief must lie in the adoption by the Government of an intelligent programme of conservation. This programme must concern itself with the uninterrupted training of physicians, nurses, and war nursing aids, in numbers sufficient to meet both military and civil needs, and with the supply of sufficient labour that is proved to be essential to the continued operation of civil hospitals. It is on these grounds that the Hospital Conference of the City of New York on June 17 passed a resolution urging the President of the United States to take such action as in his judgment would preserve the hospitals of the country from the further disruption of their organisations, and the abandonment of their indispensable public tasks.

The letter addressed to the President by the War Service Committee of the American Hospital Association covers fourteen points, which will indicate its exhaustive character. We think it well, therefore, to give the text of this important document in full.

### The A.H.A. War Service Committe's Appeal.

To the President of the United States.

June 5, 1918.

Sir,—The civil hospitals of the country, which are being gripped by destructive forces, turn to you for relief. The tendency to hospital disintegration, hardly perceptible in the first months of the war, is now rapidly gaining momentum; it can and should be halted.

There is sound military reason for asking that steps be taken to maintain hospital efficiency. An effective medical staff is indispensable to the success of the Army. Behind the Army stand the Medical and Nursing Corps; behind these the medical and nursing professions; and behind these the hospitals in which doctors and nurses must be trained.

The appointment of a national Food Administrator is the executive expression of the doctrine that "Food will win the war." Without the effective use of adequate transportation facilities we cannot win the war, and you have therefore appointed a Director-General of Railroads. Because fuel is an essential factor, a Fuel Administrator has been named. In the last analysis, it is men who will win the war, yet no Health Administrator, charged with the duty of conserving man-power, has yet appeared on the scene.

Permit me to enumerate a few of the tasks that would immediately commend themselves to a national Health Administrator in the hospital field alone :—

1. The standardisation of hospital staffs, in order that every hospital might retain in its service the actual number of men required for the care of its patients, and no more.

2. The completion of the organisation of the Volunteer Medical Service Corps, and of preparations to use the members of the Corps to fill dangerous gaps in the service of hospitals, dispensaries, and civilian communities.

3. The formulation of an economical plan for the joint use of specialists by civil hospitals and by the military hospitals which are being established to care for returned soldiers.

4. The discovery of means by which public dispensaries with dwindling staffs and limited means can be kept going.

5. Providing for the training of a sufficient number of medical students to keep up the supply of graduates needed by the Army and the civil population.

6. Procuring interns for the hospitals which have none at present.

7. The prevention of the further breaking up of the teaching staffs of nursing schools.

8. Taking adequate and timely steps to create a sufficient supply of pupil nurses.

9. The promotion of a system of legislation and a working method for the training of bedside attendants.

10. Arranging with civil hospitals for the training of a large reserve supply of volunteer war nursing aids.

11. Establishing conditions under which hospitals can successfully compete in the labour market against inflated war industries.

12. Preventing various agencies of the Government from unnecessarily hoarding medical and surgical supplies to such an extent that civil hospitals are left without surgical instruments, surgical dressings, anæsthetics, and medicines.

13. Promoting war-tax legislation which will stimulate rather than discourage donations and bequests to hospitals.

14. Providing for the relief of communities which, as a result of the war, are left without adequate medical and nursing service.

It is only by facing these problems now and by finding a speedy solution for them that the impairment of hospital and medical efficiency can be avoided. The hospitals of the country look to you with confidence for speedy and appropriate action.—Respectfully,

WAR SERVICE COMMITTEE, AMERICAN HOSPITAL ASSOCIATION. RICHARD P. BORDEN, Secretary. DANIEL D. TEST. A. R. WARNER. S. S. GOLDWATER, Chairman.

June 17, 1918.

WHEREAS, our country's increasing participation in the world-war will necessitate, in ever-growing measure, the use of physicians and nurses by the Army and Navy, the enrolment of young men as combatants, and the employment of men and women of all ages in war industries;

WHEREAS, the hospitals of New York City are experiencing grave and constantly growing difficulty in maintaining working forces adequate to their needs;

WHEREAS, the enforced closing of single departments is the prelude to the discontinuance of the work of entire institutions, unless remedial measures are instituted promptly;

WHEREAS, the future holds out no hope of relief except through the adoption by the Government of an intelligent programme of conservation, which must concern itself with the uninterrupted training of physicians, nurses, and war nursing aids, in numbers sufficient to meet both military and civic needs, and with the supply of labour which is essential to the continued operation of civil hospitals; therefore be it

**Resolved that the Hospital Conference of the City of New York urges the President of the United States to take such action as in his judgment will preserve the hospitals of the country from the further disruption of their organisations and the abandonment of their indispensable public tasks.**

